# Dapsone-induced drug reaction with eosinophilia and systemic symptoms (DRESS): the role of the primary care physician

**DOI:** 10.4314/gmj.v55i2.13

**Published:** 2021-06

**Authors:** Blessing O Akor, Bob Ukuonu, Alexander A Akor, Ojonugua A Ameh, Theresa Otu, Thairu Yunusa, Onyinye Onyeadi, Grace Lakai

**Affiliations:** 1 Department of Fam. Medicine, University of Abuja Teaching Hospital, P.M.B 228, Abuja-F.C.T. Nigeria; 2 Department of Int. Medicine, University of Abuja, P.M.B 117, Abuja, Nigeria; 3 Department of Haematology, University of Abuja, P.M.B 117, Abuja, Nigeria; 4 Department of microbiology, University of Abuja, P.M.B 117, Abuja, Nigeria; 5 Department of Pharmacy, University of Abuja Teaching Hospital, P.M.B 228, Abuja-F.C.T. Nigeria

**Keywords:** Drug Reaction, Eosinophilia, Systemic Symptoms, Dapsone, Primary Care

## Abstract

Drug reaction with eosinophilia and systemic symptoms (DRESS) is a rare but severe adverse drug reaction with debilitating morbidity and high mortality. The interest of this presentation is to report a case of dress and the role of the healthcare coordinator (family physician) in early diagnosis, prompt treatment and coordination of care. We report a case of a 32-year-old male who was wrongly commenced on Dapsone for seizure disorder from a primary health centre. He presented two months later with a month history of severe dry cough, pleurisy, intermittent breathlessness and high-grade fever. He had a generalized pruritic rash with erythema and desquamation. There was marked oedema of the face and feet with generalized lymphadenopathy. He was jaundiced with tender hepatomegaly. He had dark coloured urine and a trace of proteinuria but normal renal function. There was marked leukocytosis (35*10^9^/l) with eosinophilia (36%). His chest X-ray and viral screen for HBV, HCV and HIV were all negative. The multiple systemic presentations warranted a multidisciplinary review, and a final diagnosis of DRESS was made. His symptoms began to resolve within two days of withdrawal of the offending drug and commencement of oral corticosteroid with supportive care for his symptoms. At six weeks, there was a complete resolution of clinical features, and his laboratory parameters had returned to the baseline. Diagnosis of DRESS can be challenging, and a high index of suspicion is required. The multidisciplinary coordination of care by the first line physicians can also not be overemphasized for good outcomes.

**Funding:**

None declared

## Introduction

Drug Reaction with Eosinophilia and Systemic Symptoms (DRESS) syndrome is a severe idiosyncratic drug reaction characterized by generalized skin eruption, eosinophilia, lymphadenopathy, and end-organ damage.^1^ The terminology DRESS is an evolution of several nomenclatures coined to describe an array of presentations that characteristically follow the intake of some medications.^2^ It is a complex syndrome requiring a high index of suspicion to diagnose successfully. This is because of its multisystemic involvement. Although attempts have been made to define standardized diagnostic criteria for DRESS, yet the myriad of symptoms and it is a relatively rare condition still pose a diagnostic challenge.^3^ This also could be the reason for under-reporting. The syndrome causes a diverse array of clinical symptoms, anywhere from 2 to 8 weeks after initiating the offending drug.^1^,^2^,^4^ Mortality associated with DRESS is about 10%, and this is significantly influenced by delayed diagnosis and hence continuous exposure to the offending agent.^2^

Several medications have been implicated in the aetiology of DRESS. They include anticonvulsants (phenytoin, carbamazepine), antibiotics (particularly sulphonamides), allopurinol, abacavir, antidiuretic (frusemide) and a host of others.^2^,^3^,^5^-^7^ Dapsone, a sulphone antibiotic, has been reported as a culprit for DRESS. A systematic review by Eshki et al., it accounted for up to 17% of DRESS.^5^ The exact mechanism of action by which offending medications cause DRESS is not fully known, but genetic predisposition, among other factors, have been speculated.^4^

## Case Report

Informed consent was obtained from the patient for publication of this case.

We present a 32-year-old single male applicant who presented at the general outpatient department with a month history of severe non-productive cough, pleuritic chest pain and intermittent breathlessness. He also had a continuous high-grade fever with chills and rigours. There was a history of headache and inability to sleep, accompanied by a burning sensation on the palms and feet that began a week before the presentation. Two days before presentation, he developed a generalized pruritic rash also involving the mucous membrane of the mouth. Some swellings accompanied this on his head, neck and groin. He had severe abdominal pain mainly at the right hypochondrium and could not eat with accompanying progressive weight loss and yellowish discolouration of his eyes. Apart from dark-coloured urine, he had no other genitourinary symptom.

Our patient had a history of a seizure-like disorder about a year earlier and was placed on carbamazepine 400mg daily, which he took for about eight months. However, he was told to discontinue it and commenced on Dapsone daily by a community health extension worker. He had taken the Dapsone for about a month when his symptoms commenced. He had a previous and family history of reaction to sulphonamides.

Examination revealed an acutely ill young man with marked oedema and desquamation involving the face and lower limbs. He had generalized diffuse maculopapular exanthema. His temperature was 41°C, and he was severely jaundiced but not pale. His lips were charred with discoloured weepy, erythematous lesions at different stages. He had generalized lymphadenopathy (groin, neck, occiput and axilla). Marked epidermolysis was noted on the palms and soles with pedal oedema. He had tachycardia of 116 beats per minute with a blood pressure of 118/72mmHg and normal heart sounds. He had tachypnea of 32cycles per minute, but his chest was clinically clear. There were a soft, tender, mild hepatomegaly and mild splenomegaly too. Several investigations were done with the following results. His chest x-ray had no radiologic abnormality. The full blood count had leukocytosis of 35.7*10^9^/l with marked eosinophilia of 36% and an Erythrocyte Sedimentation Rate of 55mm/hr. There was bilirubinuria of 2+s and a trace of proteinuria, but the renal function parameters were normal.

The liver function test showed elevated bilirubin 45.6pmol/l (>2 times normal) with direct bilirubin 8.6 pmol (twice the normal).

The transaminase was 94 IU/l (>2 times the normal) with a markedly elevated alkaline phosphatase of 948 IU/l (approximately four times the normal). Hepatitis B, C and HIV screening were all negative. Abdominal USS showed mild hepatosplenomegaly with otherwise normal findings. A blood film for microfilaria was negative. Based on the above findings, the total RegiSCAR score was 7, which is definitive for DRESS.

At presentation, a provisional diagnosis of lymphoproliferative disorder with sepsis was made. He was admitted and commenced on symptomatic management with antipyretics, intravenous ceftriaxone, and antihistamines. The laboratory findings and evolving clinical state warranted consultations with several specialists (pulmonologists, haematologists, microbiologists, gastroenterologists, and dermatologists). The diagnosis of DRESS secondary to Dapsone was finally confirmed.

He was discontinued from Dapsone. The markedly elevated alkaline phosphatase warranted the use of ursodeoxycholic acid, E-45 dermatological cream, and fexofenadine were given to lubricate the dry skin and reduce the itch. He was started on oral prednisolone 10mg daily for one week then 5mg daily for another week and then tapered gradually in the third week.

Erythromycin capsules 500mg every six hours for one week, and topical hydrocortisone was also used. He began to improve almost immediately as the fever subsided, and the pruritus began to resolve.

Within two weeks of commencing treatment, oedema had resolved, and skin lesions had greatly improved. By six weeks, both clinical and laboratory findings had returned to the baseline.

## Discussion

Drug reaction with eosinophilia and systemic syndrome is a nomenclature that evolved after several terms used to describe the characteristic delayed hypersensitivity reaction that occurs with some medications.^1^ These reactions are usually preceded by a 2-8 weeks latency from when the offending medications are commenced.^1^,^2^ The index patient developed his symptoms approximately one month after he commenced Dapsone which was erroneously prescribed for an assumed seizure disorder (as neurological reviews showed no evidence of seizure). About a month later, he developed a high-grade fever followed by pleuritic chest pain, breathlessness, abdominal pain, and widespread cutaneous manifestations. These findings have been reported with DRESS.^1^,^2^,^4^,^5^ Cutaneous presentations are multiple, ranging from pruritic eruptions to diffuse maculopapular inflammatory reactions.^5^,^9^ Though several haematological disorders have been identified in DRESS; Eosinophilia is the most frequently occurring in >50% of the cases.^5^ Our patient had marked eosinophilia of 36% with a leukocytosis of 35*10^9^/l. Other haematological abnormalities like thrombocytopenia, anaemia and atypical lymphocytes have also been reported in DRESS.^1^ Another presentation which has been reported in 80% of cases is lymphadenopathy.^4^-^6^ our patient had a lymphadenopathy in the occiput, neck, axilla and groin. The abdominal presentations that have been described in the literature include abdominal pain, nausea, diarrhoea and varying hepatic involvement.^4^,^6^ Our patient had hyperbilirubinemia, elevated alanine transaminase and markedly elevated alkaline phosphatase (though not a common finding). Renal dysfunction (mostly proteinuria) was observed most often with allopurinol induced DRESS our patient had dark coloured urine and a trace of proteinuria. However, his other renal parameters were within the normal range. This might be because the renal involvement in his case was not extensive.

Eosinophilic pneumonitis, characterized by varying symptoms such as cough, dyspnea, high fever, pleuritic chest pain, peripheral blood eosinophilia and peripheral pulmonary infiltrates on radiography, have been described in some cases of DRESS.^6^

These chest findings were present in our patient, but his chest X-ray was unremarkable.

The diagnosis of DRESS syndrome is mainly clinical, and one must consider the latency period, diversity of symptoms, and exclusion of similar non-drug-induced conditions.^4^,^6^ The myriad of symptomatology as was seen in our patient where virtually every system in his body had a severe illness warrants thorough investigation to carefully rule out all possible differentials as DRESS is a diagnosis of exclusion.^6^

This will require the involvement of several specialists, not just for diagnosing but also for proper management. After confirmation of diagnosis withdrawal of the offending drug and steroid therapy remains the mainstay of treatment. But symptomatic management is also key to successful treatment.^4^,^6^,^8^

Attempts have been made to provide standardized diagnostic criteria for DRESS, and a few have emerged. The RegiSCAR (a multiparameter) scoring system is the most accurate in making a diagnosis of DRESS. A final score of less than 2 indicates no case, while a final score of greater than 5 implies a definitive case. The scoring parameters include; Reaction suspected to be drug-related, Acute rash, fever>38.5°C, Enlarged lymph nodes at a minimum of 2 sites, Involvement of at least one internal organ, Blood count abnormalities, Lymphocytes above or below normal limits, Eosinophils above the laboratory limits, Platelets below the laboratory limits.^10^ All but two of these parameters (lymphocytes and platelets abnormalities) were present in our patient giving him a final score of 7, which was definitive for DRESS,

Re-challenging with the suspected drug is considered the gold standard for drug eruptions; however, it cannot be used to confirm the culprit drug for DRESS due to the possible life-threatening consequences.^6^,^7^ In an attempt to identify a more effective diagnostic test, the patch test has been utilized but with limited usefulness, as it is drugspecific and completely negative to some DRESS causing drugs.^7^ Diagnosis of DRESS in our patient was clinical. The role of genetic predisposition in the aetiology of DRESS played out in our patient. He was on carbamazepine, a known culprit for DRESS,^4^,^8^ for eight months without symptoms. However, He developed DRESS within a month on Dapsone with a background and family history of reaction to sulphonamides.

The role of the first contact physician in coordinating care among other physicians in excluding all the possible differential diagnosis and managing the diverse systemic presentations cannot be overemphasized.

This is because a quick diagnosis with the resultant withdrawal of the offending drug and commencement of steroids is paramount for a good prognosis.^4^,^5^ This was seen in our patient, who a family physician first reviewed. He was hospitalized, stabilized, and investigated while sending consults to several specialists (pulmonologist, haematologist, microbiologist, gastroenterologist, and dermatologist) who managed the multiple systemic presentations akin to prompt patient diagnosis. Treatment is mostly supportive and symptomatic; corticosteroids are often used in organ involvement.^8^

Steroids remain the mainstay of therapy. However, a consensus is lacking regarding the optimal steroid choice, dose, route of administration, and duration of therapy.^6^ Most literature advocated supra-physiologic doses of steroids which are usually accompanied with long term complications.^5^,^6^,^8^ Our patient used near physiologic dose with excellent outcome and no complications as at five months after.

## Conclusion

DRESS syndrome is a rare but potentially life-threatening multisystem adverse drug reaction. Effective management of the condition would require prompt diagnosis, which warrants multiple disciplinary inputs, and the role of a coordinating physician (the family physician) cannot be overemphasized. Early diagnosis and prompt treatment are central to a good prognosis.

## Figures and Tables

**Figure 1a-c:**
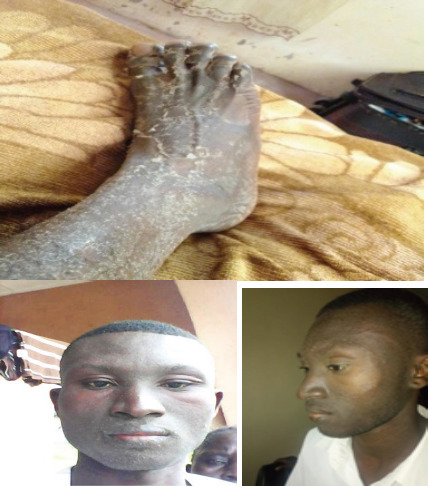
Skin manifestations showing desquamation with generalized maculopapular rash and facial oedema
